# Temperature-induced cardiac remodelling in fish

**DOI:** 10.1242/jeb.128496

**Published:** 2017-01-15

**Authors:** Adam N. Keen, Jordan M. Klaiman, Holly A. Shiels, Todd E. Gillis

**Affiliations:** 1 Division of Cardiovascular Science, School of Medicine, Faculty of Biology, Medicine and Health, University of Manchester, Manchester, M13 9NT, UK; 2 Department of Rehabilitation Medicine, University of Washington, Seattle, WA 98109, USA; 3 Department of Integrative Biology, University of Guelph, Guelph, Ontario, Canada N1G 2W1

**Keywords:** Cardiac function, Cardiac histology, Cardiac remodelling, Connective tissue, Thermal acclimation

## Abstract

Thermal acclimation causes the heart of some fish species to undergo significant remodelling. This includes changes in electrical activity, energy utilization and structural properties at the gross and molecular level of organization. The purpose of this Review is to summarize the current state of knowledge of temperature-induced structural remodelling in the fish ventricle across different levels of biological organization, and to examine how such changes result in the modification of the functional properties of the heart. The structural remodelling response is thought to be responsible for changes in cardiac stiffness, the Ca^2+^ sensitivity of force generation and the rate of force generation by the heart. Such changes to both active and passive properties help to compensate for the loss of cardiac function caused by a decrease in physiological temperature. Hence, temperature-induced cardiac remodelling is common in fish that remain active following seasonal decreases in temperature. This Review is organized around the ventricular phases of the cardiac cycle – specifically diastolic filling, isovolumic pressure generation and ejection – so that the consequences of remodelling can be fully described. We also compare the thermal acclimation-associated modifications of the fish ventricle with those seen in the mammalian ventricle in response to cardiac pathologies and exercise. Finally, we consider how the plasticity of the fish heart may be relevant to survival in a climate change context, where seasonal temperature changes could become more extreme and variable.

## Abstract (precis)

**Summary:** Thermal acclimation of some temperate fishes causes extensive remodelling of the heart. The resultant changes to the active and passive properties of the heart represent a highly integrated phenotypic response.

## Introduction

Ectothermic animals living in temperate environments can experience significant, long-term changes in ambient temperature. These seasonal fluctuations influence every level of biological function as a result of the universal effect of temperature on molecular interactions. Consequently, biochemical, physiological and biomechanical processes are all affected by changes in temperature. However, a number of ectothermic species, including some fish, remain active across the seasons. These fish species include salmonids such as rainbow trout (*Oncorhynchus mykiss*), which, depending on the strain, can remain active at temperatures ranging from ∼4 to 24°C (Anttila et al., 2014; Elliott and Elliott, 2010; Rodnick et al., 2004). Members of the minnow family, such as the zebrafish (*Danio rerio*), also have broad thermal tolerances in the wild (Cortemeglia and Beitinger, 2005; Sidhu et al., 2014) and can experience a 10°C change in temperature between winter and summer (López-Olmeda and Sánchez-Vázquez, 2011). Marine species, such as tunas, also experience temperature changes seasonally (from 11 to 24°C) associated with oceanic migrations, and acutely (>10°C change) when diving through the thermocline (Boustany et al., 2010). Although a change in temperature will affect the function of all organs, the function of the heart is especially important because of its role in moving oxygen, metabolic substrates and metabolic byproducts around the body, and therefore supporting active biological processes. Thus, many fish have mechanisms that preserve cardiac function across seasonal temperature changes.

The purpose of this Review is to examine temperature-induced structural remodelling of the ventricle in the hearts of selected fish species. We build upon excellent original work (i.e. Vornanen et al., 2005) and comprehensive reviews of cardiac plasticity in fish (e.g. Gamperl and Farrell, 2004). Importantly, here, we review changes in both the active and passive properties (see Glossary) of the fish heart following prolonged temperature change. We discuss ways in which the remodelling preserves or improves function (physiological remodelling) and ways in which the remodelling may relate to dysfunction (pathological remodelling). Indeed, one of the interesting aspects of thermal remodelling in the fish heart is that it involves changes that are similar to those observed during both physiological and pathological remodelling in mammalian hearts (see Dorn, 2007; Keen et al., 2016; Klaiman et al., 2011; Klaiman et al., 2014). We acknowledge that other aspects of fish heart function change with thermal acclimation, most notably the electrical properties. Pacemaker output can be reset, partly as a result of temperature-related changes in electrical excitability (Aho and Vornanen, 2001; Ekström et al., 2016). Electrical excitability itself is modulated by temperature-dependent shifts in ion channel densities and/or isoform switches which can vary between species and life histories (Vornanen, 2016; Badr et al., 2016).

In this Review, we focus on ventricular remodelling, primarily in two species – rainbow trout and zebrafish. Cardiac remodelling in the trout has been extensively studied and, as a cold-active species, its heart develops robust cardiac outputs (see Glossary) across a range of temperatures. We also discuss recent work on cardiac remodelling in the zebrafish – a species that has become a popular model for understanding the development and regenerative capabilities of the vertebrate heart. With >30,000 extant species of fish (Nelson, 2006), the possible remodelling phenotypes are abundant. We do not attempt to cover all of these in this Review, however, we include key studies on other fish species such as tunas, cod, flat fish and carp, where appropriate. A key aim of this Review is to show how thermal remodelling of active and passive properties work together to preserve cardiac function across temperatures. For this reason, we have divided the Review into three main sections, each addressing one of the ventricular phases of the cardiac cycle: diastolic filling, isovolumic pressure generation and ejection. Through this approach, we hope to illustrate the integrated and comprehensive nature of the thermal cardiac remodelling response.

For simplicity, we have structured the Review around observations associated with cold acclimation. Historically, responses to cold acclimation have been the main experimental interest (Bailey and Driedzic, 1990; Driedzic et al., 1996; Farrell, 1991; Haverinen and Vornanen, 2007; Keen et al., 1993, 1994; Lurman et al., 2012); however, with rising temperatures becoming a global concern, there is increasing interest in the effect of warming (Farrell et al., 1996; Farrell, 2002; Keen et al., 2016; Klaiman et al., 2011; Syme et al., 2013). Therefore, we have added a concluding section to discuss the specific implications of prolonged warm temperatures on fish heart function.

Glossary **Active properties of the heart**

Properties that affect muscle contraction, including rate of cross-bridge cycling and sensitivity to Ca^2+^.

**Bradycardia**

A reduction in the rate of cardiac contraction.

**Cardiac contractility**

Ability of heart to contract and generate force when stimulated by Ca^2+^.

**Cardiac myofilaments**

Primarily composed of the actin thin filament and myosin thick filament and responsible for force generation in striated muscle.

**Cardiac output**

Blood volume pumped by the heart per unit time, calculated as the product of contraction Hz and stroke volume.

**Cardiac stiffness**

Ability of the heart to resist stretching, determined by both the active and passive properties of the muscle. Inverse of compliance.

**Cardioplegic**

Reduction in cardiac contractility.

**Chamber compliance**

Inverse of stiffness, can be measured as the change in pressure for a given change in volume.

**Inotropic effects**

Affecting the force of contraction.

**Passive properties of the heart**

Non-contractile properties that affect the stiffness of the heart and influence the ability of the heart to relax and fill with blood between heartbeats. This is affected by collagen composition and the sarcomere protein titin.

***Q*_10_ effects**

The change in rate of biochemical reaction that occurs with a 10°C change in temperature.

**Ventricular trabeculae**

Discrete bundles or sheets of muscle within the spongy myocardium of fish.

## Acute temperature change and cardiac function

### Acute effects on whole heart function

Acute temperature change (minutes to hours) directly influences physiological processes in fish through *Q*_10_ effects (see Glossary) on reaction rates. As the temperature drops, the heart becomes bradycardic (see Glossary; Keen et al., 1993), which is largely due to a greater diastolic duration, with systolic duration less affected (Badr et al., 2016). The greater diastolic duration acts to maintain cardiac output by increasing filling time, which can lead to an increase in stroke volume even though cardiac contractility (see Glossary), force production and cycle frequency are reduced at lower temperatures (Shiels et al., 2002; Vornanen et al., 2005). Changes in cycle frequency (i.e. heart rate; as reviewed by Vornanen, 2016) directly alter cellular processes within the heart, independent of temperature. While this is of prime importance to cardiac function, this Review focuses on the force-generating capacity of the myocardium rather than cycle frequency. Changes in cardiac force are often the inverse of rate changes and compensate (at least partially) for the direct effect of temperature in altering cycle frequency. Acute cooling also increases blood viscosity, which directly affects vascular resistance and increases cardiac load (Graham and Farrell, 1989; Graham and Fletcher, 1985). Although these effects of acute temperature are detrimental to contractile function, chronic exposure results in compensatory changes that limit their consequences for cardiac output, as discussed later in this Review.

### Acute effects on the myofilaments

**Fig. 1.**
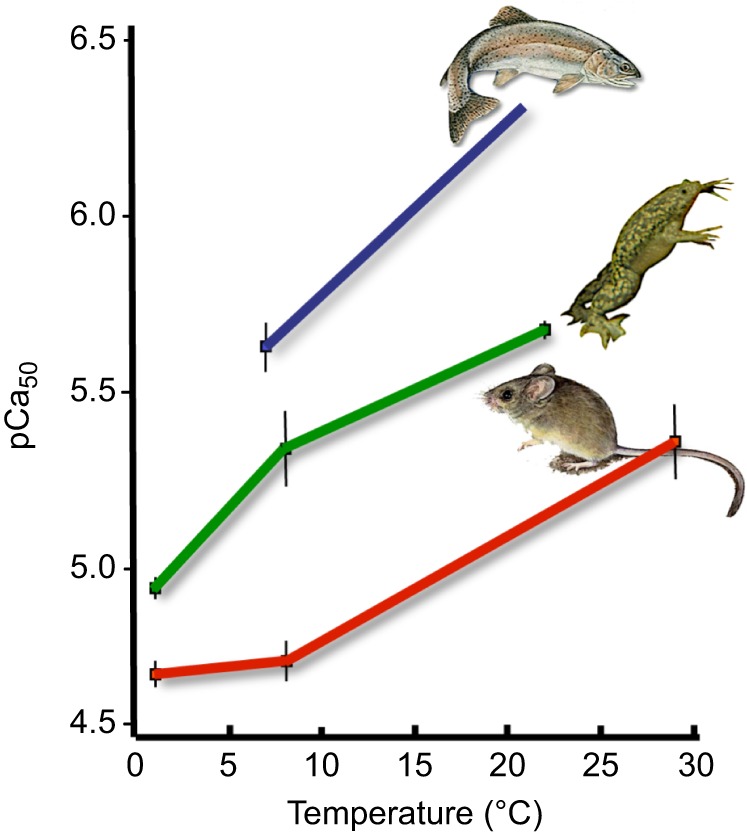
**Ca^2+^ sensitivity of force generation by skinned ventricular fibres over a range of temperatures.** pCa_50_ is the Ca^2+^ concentration required to generate half-maximum force. When compared at the same temperature, trout ventricular fibres require 10 times less Ca^2+^ than those from the rat to generate the same amount of force. Figure adapted from Churcott et al. (1994).

An acute decrease in the temperature of the vertebrate heart, including those from mammals and fish, impairs contractile function, as the thin filaments in cardiac muscle have a reduced sensitivity to Ca^2+^ at lower temperatures, resulting in a loss of force-generating capacity (Churcott et al., 1994; Harrison and Bers, 1990; Stephenson and Williams, 1985). See Box 1 for an explanation of the Ca^2+^-mediated activation of cardiac contraction. The cold-associated decrease in Ca^2+^ sensitivity in cardiac muscle has been reported in a variety of animals, including trout, frogs, mice, rats, rabbits, ferrets and ground squirrels (Churcott et al., 1994; Harrison and Bers, 1989; Liu et al., 1993, 1990). Studies by Gillis et al. (Gillis et al., 2005, 2000, 2003b; Gillis and Tibbits, 2002) show that this decrease in Ca^2+^ sensitivity following an acute reduction in temperature is due to a decrease in the Ca^2+^ affinity of cardiac troponin C, which is the Ca^2+^-activated trigger for the muscle (see Box 1). Although the cardiac muscle of trout and mammals behaves in a similar way in response to reduced temperatures, trout myofilaments (see Glossary) have several characteristics that allow the heart to remain functional over a range of physiological temperatures, including low temperatures. Churcott et al. (1994) demonstrated that trout cardiac actin-myosin ATPase activity was more Ca^2+^ sensitive than that from rats when compared at their respective physiological temperature and pH (7°C, pH 7.2 vs 37°C, pH 6.78 for trout and rat, respectively). Moreover, the authors found that the Ca^2+^ concentration required by trout cardiac muscle preparations to reach half maximal tension was approximately one-tenth that of rat cardiac tissue when tested at the same experimental temperature (Fig. 1). This higher Ca^2+^ sensitivity of the trout cardiac tissue is believed to be one mechanism that helps to offset the cardioplegic effects (see Glossary) of cold on the trout heart (Blumenschein et al., 2004; Gillis et al., 2003a). These interactions will be discussed further in the section ‘Myofibril remodelling’.

Box 1. Ca^2+^-mediated activation of the heart Ca^2+^ is responsible for initiating and regulating the contraction of striated muscle. Following the firing of the sinoatrial node, also known as the cardiac pacemaker, cellular membranes of cardiac myocytes in the heart are depolarized, which causes the L-type Ca^2+^ channels to open. As a result, Ca^2+^ enters the cell and can interact directly with the myofilaments. Ca^2+^ influx can also activate the ryanodine receptors (RyRs) located in the membrane of the sarcoplasmic reticulum (SR). The SR is an organelle that stores and releases Ca^2+^ in the myocyte. The activation of the RyRs causes the release of Ca^2+^ from the SR into the cytosol in a process called Ca^2+^-initiated Ca^2+^ release (CICR). CICR is vital for the contraction of mammalian hearts but less so for fish hearts, as extracellular Ca^2+^ influx delivers sufficient Ca^2+^ to the myofilaments in most fish species (see Shiels and Galli, 2014). The increase in cytosolic Ca^2+^ activates the actin thin filament when Ca^2+^ binds to the troponin (Tn) complex through cardiac troponin C (cTnC). Ca^2+^ binds to a binding site in the N-terminus of the protein, which initiates a conformational change in the molecule that triggers a series of further conformational changes through the other component proteins of the thin filament, leading to the exposure of a myosin-binding site on the surface of actin (see Gillis and Tibbits, 2002). As a result, a myosin head binds to the actin thin filament, resulting in the formation of a cross-bridge. The cross-bridge generates force with the hydrolysis of ATP, and the myosin head flexes. The formation of force-generating cross-bridges along the contractile element leads to the shortening of the sarcomere and the contraction of the muscle during systole. As a result, blood is pumped out of the heart. For the heart to relax, Ca^2+^ is pumped back into the SR through the SR Ca^2+^-ATPase or out of the cell through the Na^+^/Ca^2+^ exchanger, causing cytosolic Ca^2+^ concentrations to decrease. This causes Ca^2+^ to disassociate from the actin thin filament, resulting in the inhibition of further cross-bridge formation. Inactivation of the cross-bridge cycle enables the myocardium to relax and then fill with blood during diastole.

### Acute effects on ion channel flux and the action potential

**Fig. 2.**
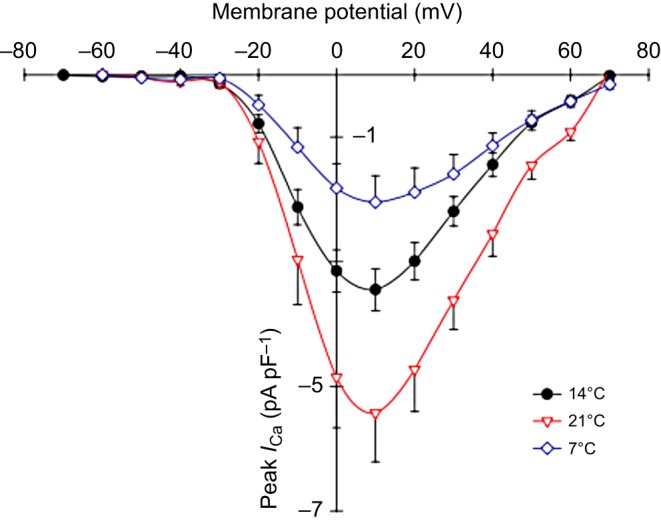
**Trans-sarcolemmal Ca^2+^ flux varies in trout cardiac myocytes with acute temperature changes.** Acute reductions in temperature reduce Ca^2+^ flux through L-type Ca^2+^ channels in rainbow trout atrial myocytes. All values are means±s.e.m. The values for *I*_Ca_ (pA) are normalized from the measured cell capacitance to give the value in pA pF^−1^. Figure adapted from Shiels et al. (2000).

Acute cooling reduces the flux of Ca^2+^ (*I*_Ca_, the Ca^2+^ current) through voltage-gated Ca^2+^ channels into the myocyte (Fig. 2), which can directly reduce the contractility of the heart at cold temperatures. This is because *I*_Ca_ is the primary source of the activating Ca^2+^ that triggers cross-bridge cycling. In fish species that utilize intracellular Ca^2+^ stores of the sarcoplasmic reticulum (SR) in the activation of muscle contraction [e.g. rainbow trout (Hove-Madsen and Tort, 1998; Shiels and White, 2005); burbot (*Lota lota*; Shiels et al., 2006b); yellowfin tuna (*Thunnus albacares*; Shiels et al., 1999); bluefin tuna (*Thunnus orientalis*; Shiels et al., 2011); Box 1], the reduction in *I*_Ca_ has a cascading effect: a reduced amplitude of *I*_Ca_ reduces the trigger for SR Ca^2+^ release, thus reducing the amount of Ca^2+^ available to interact with the myofilaments and initiate cross-bridge cycling.

Some of the direct effects of reduced *I*_Ca_ during cooling can be offset by other temperature-induced changes in the electrical properties of the heart. For example, acute cooling increases the duration of the ventricular action potential [e.g. rainbow trout (Shiels et al., 2000); bluefin tuna (Galli et al., 2009); pink salmon (*Oncorhynchus gorbuscha*) (Ballesta et al., 2012)]. This allows more time for Ca^2+^ influx during the action potential plateau, possibly on the *I*_Ca_ window current (see Vornanen, 1998), which occurs when L-type Ca^2+^ channels that have inactivated reopen during the action potential plateau. As the action potential duration is extended during cooling, it can allow a larger *I*_Ca_ window current. It is important to note that in some species, like bluefin tuna, the drop in Ca^2+^ influx during cooling is not completely compensated for by the increased action potential duration. In these hearts, adrenaline, which is thought to be released during dives into cold water, augments Ca^2+^ influx through voltage-gated ion channels. This increased Ca^2+^ influx combines with a prolonged action potential duration to restore force-generating Ca^2+^ flux into the myocytes during temperature changes of >10°C (Shiels et al., 2015).

Although this trade-off between action potential duration and Ca^2+^ influx can maintain adequate Ca^2+^ influx to allow the fish to cope with short-term changes in temperature, it is less effective during prolonged thermal acclimation. Indeed, during chronic (days to weeks) cold exposure there is a remodelling of potassium (K^+^) channel expression that serves to reverse the increase in action potential duration. This is important, as a prolonged action potential can be pro-arrhythmic and also may compromise electrical restitution (the recovery of an action potential as a function of the diastolic interval). These temperature-induced alterations in the ion channels of the fish heart are discussed in detail in a recent review (Vornanen, 2016). Together, the effects of an acute decrease in temperature on electrical and mechanical function lead to a reduction in the force of cardiac muscle contraction (inotropic effects; see Glossary), illustrating the need for temperature-dependent remodelling to preserve the active pumping properties of the fish heart during chronic temperature change.

### Acute effects on the diastolic properties of the heart

An acute temperature change also influences the resting, non-force generating properties of the heart by affecting the passive properties of the myocardium. For example, an increase in temperature decreases the contribution of viscous tension, viscoelastic tension and elastic tension to cardiac stiffness (see Glossary), resulting in decreased passive stiffness (Mutungi and Ranatunga, 1998). Together, the changes in the non-force-generating properties of the muscle caused by a change in physiological temperature represent a potential challenge for the maintenance of normal cardiac function. It is, therefore, not surprising that factors which contribute to the passive properties of the heart, such as collagen content and composition, are modified in response to thermal acclimation (Keen et al., 2016; Klaiman et al., 2011; Johnson et al., 2014).

## Cardiac remodelling following chronic temperature change

Evidently, acute temperature change is a challenge for maintained cardiac function in fishes. Thus, prolonged temperature change results in remodelling of all aspects of cardiac function. For example, in relation to the direct effects of acute cooling discussed above, cold acclimation results in an increase in basal heart rate (Haverinen and Vornanen, 2007; Keen et al., 1993; Lurman et al., 2012), maximum stroke volume (Driedzic et al., 1996; Farrell, 1991; Lurman et al., 2012), maximum power output (Bailey and Driedzic, 1990; Lurman et al., 2012) and maximum cardiac output (Lurman et al., 2012), as well as a greater sensitivity to β-adrenergic stimulation (Keen et al., 1993). For excellent reviews of energetics and electrical activity associated with thermal acclimation in fishes see Driedzic and Gesser (1994), Vornanen et al. (2002) and Vornanen (2016). Below, we focus on the active and passive changes associated with structural remodelling of the fish heart.

### Phase 1 – Diastolic filling of the ventricle

**Fig. 3.**
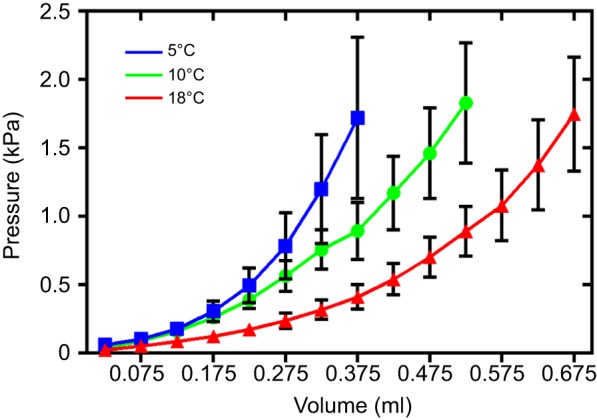
**Thermal remodelling of ventricular compliance in the rainbow trout.** *Ex vivo* pressure–volume relationships show increased passive stiffness of the whole ventricle following cold acclimation (5°C) compared with controls (10°C), and increased compliance following warm acclimation (18°C). Data points show the means±s.e. All lines are significantly different from each other, assessed via GLM. Figure is adapted from Keen et al. (2016).

The first stage of the cardiac cycle is diastolic filling. As the ventricle relaxes, ventricular pressure decreases. When ventricular pressure drops below atrial pressure, the atrioventricular valve opens and blood flows from the atrium into the ventricle. This phase of the cardiac cycle is known as isovolumic relaxation, and it lasts from the time when the atrioventricular valves open until they close again. Ventricular pressure and, therefore, diastolic filling volume are largely determined by cardiac preload, which is determined by venous pressure and atrial systole. The sinus venosus and atrium are larger than the ventricle and act as reservoirs by modulating the volume of blood entering the heart (Farrell, 1991). To maintain correct diastolic function, the ventricle must be compliant enough to allow sufficient filling, but also needs to be strong enough to withstand the haemodynamic stress of pumping a large volume of blood. Passive tension describes the resistance of a cardiac chamber to diastolic filling and, therefore, plays a role in the Frank–Starling response of the heart (Shiels and White, 2008), where an increase in end-diastolic volume results in an increase in systolic contraction and stroke volume. In rainbow trout, passive stiffness of the whole ventricle increases following cold acclimation, as shown by generating *ex vivo* pressure–volume relationships (Fig. 3) (Keen et al., 2016). Functionally, these decreases in chamber compliance (see Glossary) may be cardioprotective, by providing support to the cardiac wall to counteract the increased haemodynamic stress encountered during high cardiac load. However, excessive stiffening of the myocardium has been shown in mammals to reduce diastolic filling and, in severe cases, can lead to diastolic dysfunction (Collier et al., 2012). It is currently unclear how increased diastolic stiffness affects *in vivo* diastolic filling in fish. These features are discussed in more detail below.

#### Stiffness, compliance and the extracellular matrix

The end-diastolic pressure–volume relationship describes myocardial relaxation. This relationship, and therefore cardiac compliance, is influenced at the organ level by the pericardium and by the geometry and thickness of the ventricular walls. In fish, the ratio of spongy to compact tissue is also likely to contribute to cardiac compliance, with compact myocardium being stiffer than spongy myocardium. Historically, ventricular wall thickness and connective tissue content were thought to be the dominating factors driving ventricular compliance; however, there is now evidence to suggest that there are important contributing roles for many extracellular and intracellular mechanisms. In fish hearts, it is likely that a combination of factors determine overall passive stiffness.

The main components of the cardiac extracellular matrix (ECM) are the interstitial fibrous proteins, collagen and elastin and glycosaminoglycans, which connect to ECM proteins to form proteoglycans (Cleutjens and Creemers, 2002; Fomovsky and Holmes, 2010). The elastic elements of the ECM (collagen and elastin) provide structure and support to the chamber walls and are, therefore, central to the overall passive tension of the ventricle (Katz, 2006). Matrix proteins also surround individual myocytes, muscle bundles and blood vessels, forming a complex structural network of interstitial matrix and basement membrane (Sanchez-Quintana et al., 1995). Together, this network of proteins helps to maintain the structural integrity of the heart while also enabling – and controlling – the distensibility (i.e. the fold change in cardiac compliance) of the tissue.

**Fig. 4.**
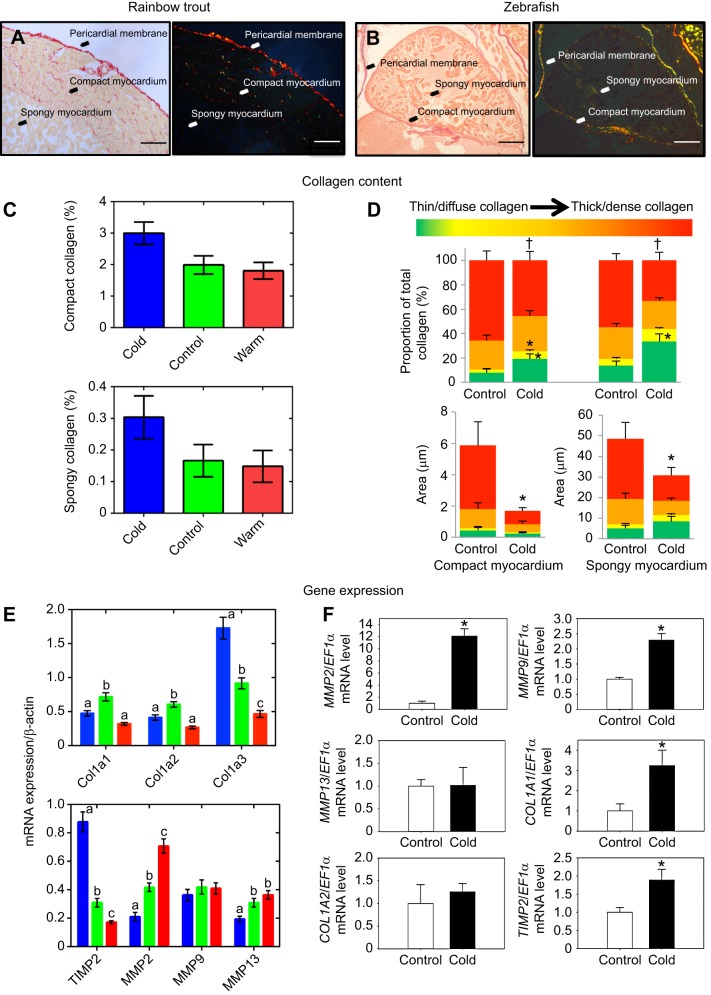
**Thermal remodelling of ventricular collagen in rainbow trout and zebrafish.** Representative bright-field (left) and polarised-light (right) micrographs of control (A) rainbow trout and (B) zebrafish ventricular tissue sections stained with PicroSirius Red, which allows semi-quantification of fibrillar collagen content. Cold acclimation causes an increase in ventricular collagen content in (C) rainbow trout, but (D) a decrease in thick collagen fibres in the zebrafish ventricle. (E) Increased ventricular collagen content in rainbow trout is associated with increased mRNA expression of collagen-promoting genes (5°C; blue), compared with control (10°C; green), whereas warm acclimation (18°C; red) is associated with an increase in mRNA expression of collagen-degrading genes. (F) Following cold acclimation, zebrafish ventricles show an increase in mRNA expression of collagen-regulatory genes, suggesting increased collagen turnover. In the zebrafish experiment fish were maintained at 27°C (Control) or acclimated to 20°C (cold). All data are means±s.e. Letters and symbols indicate significant differences. Figures modified from Johnson et al. (2014) and Keen et al. (2016). Scale bars: 100 μm.

Collagen is the most common structural protein in the ECM (Fomovsky and Holmes, 2010). It forms stiff fibres that support and maintain the alignment of myocytes by bearing wall stress. At high chamber volumes, the collagen fibres become stiff and straight to resist overexpansion and damage to myocytes (Fomovsky and Holmes, 2010). In mammals, chronic increases in cardiac load are often associated with increased myocardial collagen deposition, which allows the heart to resist the increased haemodynamic stress. Collagen also increases the passive stiffness of the chamber wall, so excessive fibrosis of the myocardium can reduce chamber compliance and chamber distensibility, which can have implications for diastolic filling (Collier et al., 2012). In the fish heart, collagen can be identified using PicroSirius Red staining, and it is visible in both the compact and spongy myocardium (Fig. 4A,B). In rainbow trout, myocardial fibrillar collagen content (Keen et al., 2016; Klaiman et al., 2011) and/or connective tissue content (Keen et al., 2016; Klaiman et al., 2011) increases ∼1.7-fold and ∼3.5-fold, respectively, following cold acclimation (Fig. 4C), which is likely to protect the myocardium from the increased haemodynamic stress of pumping cold viscous blood. However, the opposite response has been observed in zebrafish, where there is significantly less thick collagen fibres in the hearts of fish acclimated to 20°C compared with those acclimated to 28°C (Fig. 4D) (Johnson et al., 2014). One potential explanation for these opposing responses is related to the difference in blood pressure between zebrafish and trout. Adult zebrafish weigh between 0.3 and 1.0 g (Fuzzen et al., 2010) and measurements completed by Hu et al. (2001) indicate that peak ventricular pressure in these fish is 3 mmHg. Meanwhile, the blood pressure of ∼750 g trout is approximately 50 mmHg (Clark and Rodnick, 1999). This suggests that there is less pressure to inflate the zebrafish heart. Therefore, an increase in the stiffness of the zebrafish myocardium caused by an acute decrease in temperature would make it more difficult for the lacunae in the zebrafish heart to fill with blood during diastole. Further work is required to compare how cold acclimation influences the passive stiffness of trout and zebrafish hearts. However, recent work by Lee et al. (2016) using high-resolution echocardiography demonstrates that cold acclimation of zebrafish does not alter the early peak velocity:atrial peak velocity (E/A) ratio (i.e. the ratio of early ventricular filling, where blood flows into the ventricle solely due to pressure gradient, to ventricular filling aided by atrial contraction, which is the second phase of atrial filling), indicating that there was no loss of diastolic function. This study also demonstrated that cold-acclimated fish had a slower isovolumetric contraction time compared with warm-acclimated fish when measured at 18°C (Lee et al., 2016). This suggests that cold-acclimated fish show improved ejection, and that the zebrafish is able to effectively compensate for the influence of low temperature on cardiac function following cold acclimation.

Myocardial collagen content reflects a balance between collagen deposition and degradation. Collagen degradation is regulated by matrix metalloproteinase (MMPs), and the gelatinase activity of MMPs is regulated by tissue inhibitors of MMPs (TIMPs). Increased enzymatic activity of TIMPs inhibits collagen degradation by MMPs, and is associated with increased collagen deposition. With cold-induced ventricular hypertrophy and fibrosis in rainbow trout, myocardial expression of MMP2 and MMP13 mRNA is downregulated (Keen et al., 2016), and there is an associated upregulation of TIMP2 mRNA transcripts (Fig. 4E) (Keen et al., 2016). Conversely, cold acclimation of zebrafish – which causes a decrease in collagen content and in the proportion of thick collagen fibres in the compact myocardium – is associated with an increase in the level of gene transcripts for MMP2 and MMP9 in the heart (Fig. 4F) (Johnson et al., 2014). This suggests that there is an increase in collagen turnover that would result in the observed changes in collagen content (Johnson et al., 2014), and is further evidence that MMPs play a role in regulating collagen content in the fish heart during thermal acclimation.

The predominant fibrillar collagen in cardiac tissue is collagen I, followed by collagen III (Eghbali and Weber, 1990). Fibrillar collagen molecules are made by super-coiling three alpha amino acid chains into an α-helix. In mammals, collagen I is composed of two type 1 (α1) and one type 2 (α2) subunits. However, in collagen I of bony fishes, one of the α1 chains is replaced with a type 3 (α3) subunit (Saito et al., 2001). Keen et al. (2016) showed this fish-specific α3 chain is upregulated 1.4-fold with the cold-induced fibrosis observed in the trout heart. Interestingly, the α3 chain reduces the denaturation temperature of the collagen I molecule and makes it more susceptible to degradation by MMP13 (Saito et al., 2001), which may explain the transient nature of cardiac fibrosis in trout following thermal acclimation. Comparatively, in mammals, total cardiac connective tissue increases of ∼1.6-fold are considered to be a pathological condition that stiffens the myocardium, which is often associated with ∼1.3- to 2.1-fold increases in the ratio of type I:type III collagen – type I collagen is less extensible than type III (Jalil et al., 1988, 1989; Marijianowski et al., 1995; Pauschinger et al., 1999). Such changes are common, and permanent, in the hearts of patients suffering from cardiac hypertension, dilated cardiomyopathy or chronic congestive heart failure, and they contribute to the associated diastolic dysfunction and eventual heart failure (Jalil et al., 1988, 1989; Marijianowski et al., 1995; Pauschinger et al., 1999). The ability of fish species, including the zebrafish and trout, to regulate myocardial collagen content in response to changes in physiological conditions suggests that fish show greater cardiac phenotypic plasticity than mammals.

#### Intracellular contribution to stiffness and compliance

At the myocyte level, cardiac compliance during diastolic filling is influenced by a number of features. Firstly, the amount and speed of Ca^2+^ removal from the cytoplasm by the SR and the Na^+^/Ca^2+^ exchanger alters stiffness and compliance through residual active tension. The Ca^2+^ affinity of troponin and the dissociation of contractile proteins once Ca^2+^ has dissociated from troponin (Katz, 2006) influences this relationship. Secondly, passive stiffness of the cytoskeleton and of sarcomeric proteins such as titin plays a large role in determining overall myocyte stiffness and compliance (Granzier et al., 1996; Horowits et al., 1989; Shiels and White, 2008; Watanabe et al., 2002). Titin is a giant sarcomeric protein that runs from the Z-line through to the M-line (Helmes et al., 1996; Linke, 2008; Linke et al., 1996; Peng et al., 2007; Wu et al., 2000). Two titin isoforms exist in the vertebrate adult heart: a shorter and stiffer N2B isoform and a longer and more compliant N2BA isoform (Cazorla et al., 2000; Patrick et al., 2010). The ratio of the two isoforms modulates titin-based passive tension (Cazorla et al., 2000; Fukuda et al., 2005; Linke, 2008; Trombitas et al., 2001). In addition, phosphorylation of the N2B element by protein kinase A (PKA) or protein kinase G (PKG) can decrease passive force (Krüger and Linke, 2009). Cardiac output in the rainbow trout heart can be modulated by up to 3-fold increases in stroke volume. Therefore, it is perhaps unsurprising that rainbow trout ventricular myocytes have a higher ratio of the compliant N2BA isoform to the stiffer N2B isoform compared with a rat myocyte (Patrick et al., 2010). However, passive tension remains higher in a fish myocyte than a rat myocyte due to a lower level of titin phosphorylation, which may explain the large Frank–Starling response in fish hearts (Patrick et al., 2010).

At present, the effect of temperature acclimation on the intracellular structure and titin remodelling in the fish heart is not known. In mammals, the expression of specific titin isoforms shows plasticity, with the changing haemodynamics that occur during cardiac growth altering titin ratios, but little is known about the mechanism (Linke, 2008). The ratios of titin isoforms have been suggested to shift to compensate for cardiac fibrosis by increasing the expression of the compliant N2BA isoform (Neagoe et al., 2002). However, increased compliance of titin may reduce systolic function via the Frank–Starling mechanism because of reduced titin spring activity (Linke, 2008). In fish, the titin isoform ratio is also likely to be an important feature for determining the passive properties of the fish heart. Keen et al. (2016) demonstrated this in rainbow trout by measuring micromechanical stiffness of ventricular tissue sections with atomic force microscopy. Cold acclimation increased micromechanical stiffness by ∼1.9-fold (to ∼0.85 MPa), which is comparable to the stiffness recorded in scarred mammalian myocardium following myocardial infarction (∼0.8 MPa) (Hiesinger et al., 2012). Furthermore, cumulative frequency curves showed an even distribution of tissue stiffness, suggesting that tissue stiffness was increasing evenly across the tissue rather than due to specific increases in the stiffness of the structural components of the tissue, such as fibrillar collagen. Future studies should aim to understand the changes in the intracellular structure of the fish myocyte that occur with temperature acclimation and how these contribute to the overall changes in passive tension of the fish ventricle.

#### Cardiac hypertrophy

In mammals, wall thickness is known to affect passive stiffness of the ventricle, therefore hypertrophy (muscle growth) or atrophy (muscle loss) of the ventricle may influence the diastolic filling phase of the cardiac cycle. In the mammalian heart, hypertrophy is initiated by increased cardiac load caused by physiological stressors, including aerobic exercise and pregnancy, or a pathological condition, such as a myocardial infarction or hypertension (Dorn, 2007; Dorn et al., 2003). The elevated biomechanical strain of chronic pressure or volume overload causes increased tension of the heart wall, which triggers increased mRNA production and protein synthesis leading to cellular hypertrophy and increased connective tissue (Bishop, 1990; Nadal-Ginard et al., 2003). Capillary growth is vital to provide the growing cardiac muscle with a sufficient supply of oxygen and nutrition; thus, the secretion of angiogenic factors, such as vascular endothelial growth factor (VEGF) is also observed (Weber and Janicki, 1989).

A number of studies have shown increased ventricular mass (relative to body mass) in fish following cold acclimation (Aho and Vornanen, 1998; Driedzic et al., 1996; Farrell et al., 1988; Kent et al., 1988; Klaiman et al., 2011; Vornanen et al., 2005). The increased ventricular mass is mainly attributed to an increase in myocyte size, suggesting it is a physiological hypertrophic response, in the spongy layer (Aho and Vornanen, 1998; Driedzic et al., 1996; Keen et al., 2016; Klaiman et al., 2011; Vornanen et al., 2005). However, some studies suggest that myocyte hyperplasia (increase in cell numbers) accounts for around 20% of myocardial growth, in addition to hypertrophy (Farrell et al., 1988; Keen et al., 2016; Sun et al., 2009). The mRNA expression of VEGF is upregulated during cold acclimation, suggesting an increased blood supply to the compact layer (Jørgensen et al., 2014; Keen et al., 2016). This hypertrophic response upon cold acclimation, along with the increase in cardiac connective tissue, indicates that changes in physiological conditions can elicit a significant phenotypic response as the heart continues to function.

### Phase 2 – Pressure generation

The second stage of the cardiac cycle is pressure generation. Following ventricle filling, the ventricular myocardium starts to contract isometrically, building up pressure within the ventricle, which closes the atrioventricular valve. An increase in end-diastolic volume results in an increase in systolic contraction and stroke volume (Frank–Starling response). At the cellular level, an increase in pressure during ventricle loading stretches the myocytes in the ventricle, increasing sarcomere length (SL) and, thus, changing the force of contraction (reviewed in Shiels and White, 2008). Mammalian cardiac myocytes show an increase in the force of contraction with an increase in SL until a peak of ∼2.2 μm (Gordon et al., 1966); however, Shiels et al. (2006a) have demonstrated that the active force of contraction in trout cardiac myocytes increases until an SL of 2.6 μm. Since the trout heart has a high ejection fraction volume (>80%; Franklin and Davie, 1992), this would allow the ventricle to be stretched to a greater extent, and as a result, allow for greater diastolic filling and increased strength of contraction. These factors are critical to the regulation of cardiac output via the Frank–Starling mechanism in fish (Shiels et al., 2006a).

#### Myofibril remodelling

Force is produced in striated muscle by the cycling of cross-bridges between the actin thin filaments and myosin thick filaments. This reaction, initiated by Ca^2+^ binding to the thin filament, results in muscle contraction. One mechanism for regulating contractile function in skeletal or cardiac muscle in the face of an environmental stressor is to express an isoform of a protein that is better suited for a particular physiological condition. For example, Crockford and Johnston (1990) demonstrated that cold acclimation of carp resulted in the expression of a unique myosin light chain (MLC) in skeletal muscle and also increased the expression of MLC-1 while decreasing the expression of MLC-3. Previous work by Vornanen (1994) has demonstrated that one isoform of MHC is expressed in the skeletal muscle of carp in winter but that two isoforms are expressed in the same muscle in summer. These changes in protein expression correlate with altered myocyte contractility (Crockford and Johnston, 1990; Vornanen, 1994). In the trout heart, cold acclimation has been shown to alter the gene transcript levels for different isoforms of cardiac myofilament proteins. More specifically, Genge et al. (2013) identified transcripts for two isoforms of TnC in the trout heart that are modulated by cold acclimation. Troponin C (TnC) is the Ca^2+^-activated trigger that initiates myocyte contraction (Box 1), and previous studies have demonstrated that manipulation of the isoform working in the muscle can alter contractile function (Gillis et al., 2005). In addition, Alderman et al. (2012) demonstrated that the trout heart expresses the gene transcripts for seven different TnI isoforms, and that the abundance of four of these changes with cold acclimation. There are considerable differences within the sequences of the seven TnI isoforms found in trout heart (Alderman et al., 2012), which likely result in differences in the functional properties of the protein. If the changes in TnI transcript abundance translate into changes in the complement of protein isoforms present in the muscle, this would potentially alter the Ca^2+^ sensitivity or the kinetics of contraction. Such a strategy may be utilized to maintain contractile function in the trout heart with cold acclimation.

**Fig. 5.**
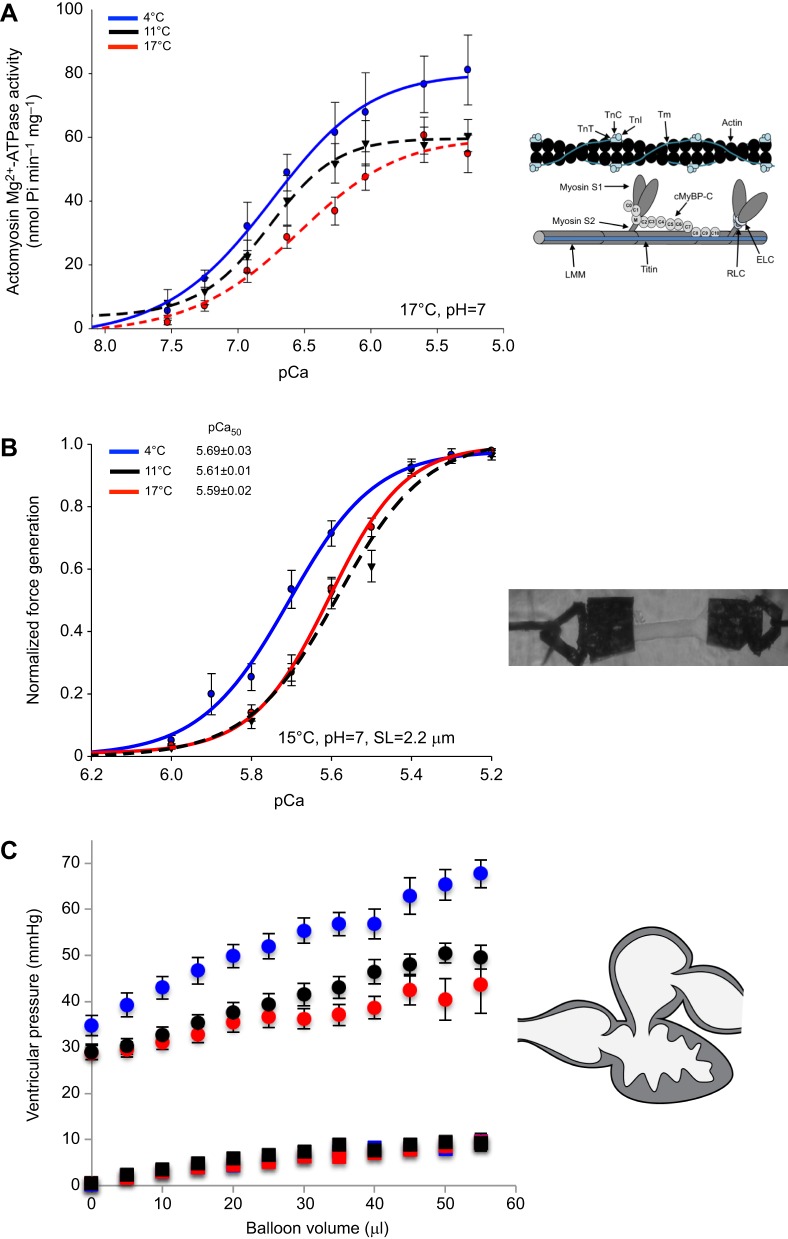
**Cardiac contractile properties of trout acclimated to 4°C, 11°C and 17°C.** (A) The maximal activity of actomyosin Mg^2+^-ATPase isolated from ventricles is higher in preparations from cold-acclimated trout than those from warm-acclimated trout when measured at 17°C. (B) The Ca^2+^ sensitivity of force generation by cardiac trabeculae from trout acclimated to 4°C (blue line) is greater than that of trabeculae from trout acclimated to 11°C (black line) or 17°C (red line) when measured at 15°C. pCa_50_ is the pCa at half-maximum force. SL, sarcomere length. (C) Developed pressures at ventricle volumes greater than baseline are higher for the 4°C acclimated (blue symbols) fish than those for the 11°C (black symbols) and 17°C (red symbols) acclimated fish. Circles indicate ventricular developed pressures, while squares indicate diastolic pressures. All data are means± s.e. Figures modified from Klaiman et al. (2011) and Klaiman et al. (2014). The images on the right of the panels are: (A) a schematic of a thick and thin filament inside a cardiac myofilament; (B) a micrograph of a trout cardiac myofilament preparation attached to a force transducer and servo motor via aluminium clips; and (C) a schematic of a trout heart.

Phosphorylation of key regulatory proteins – including cardiac troponin I (cTnI), cardiac troponin T (cTnT) and myosin binding protein C (MyBP-C) – can modulate myofilament function in the vertebrate heart (reviewed by Shaffer and Gillis, 2010). In the mammalian heart, these proteins can be targeted by protein kinase A (PKA) or protein kinase C (PKC) following β-adrenergic or α-adrenergic stimulation, respectively (Shaffer and Gillis, 2010). The resultant functional changes that follow PKA phosphorylation in the mammalian heart include a decrease in the Ca^2+^ sensitivity of force generation, increased kinetics of Ca^2+^ activation and a decrease in force generation (Chandra et al., 1997; Dong et al., 2007). Using a chemically skinned myofilament preparation from trout hearts, it has been shown that PKA phosphorylation decreases cross-bridge cycling and maximal force generation (Gillis and Klaiman, 2011). Interestingly, cold acclimation of trout results in an increase in the maximal rate of the cardiac actomyosin-ATPase activity (Klaiman et al., 2014; Yang et al., 2000) (Fig. 5A), an increase in the Ca^2+^ sensitivity of force generation by skinned ventricular trabeculae (see Glossary; Klaiman et al., 2014) (Fig. 5B) as well as an increase in the magnitude and rate of pressure generation by the isolated heart (Fig. 5C) (Klaiman et al., 2014). This indicates that the heart functions better with cold acclimation (Klaiman et al., 2014). Quantification of phosphorylation of the myofilament proteins in the cold-acclimated hearts demonstrates a decrease in the phosphorylation of cTnT, slow skeletal TnT and MyBP-C. This suggests that the changes in myofilament function are due, at least in part, to post-translational changes in the myofilament regulatory proteins (Klaiman et al., 2011, 2014).

#### Cardiac morphology

**Fig. 6.**
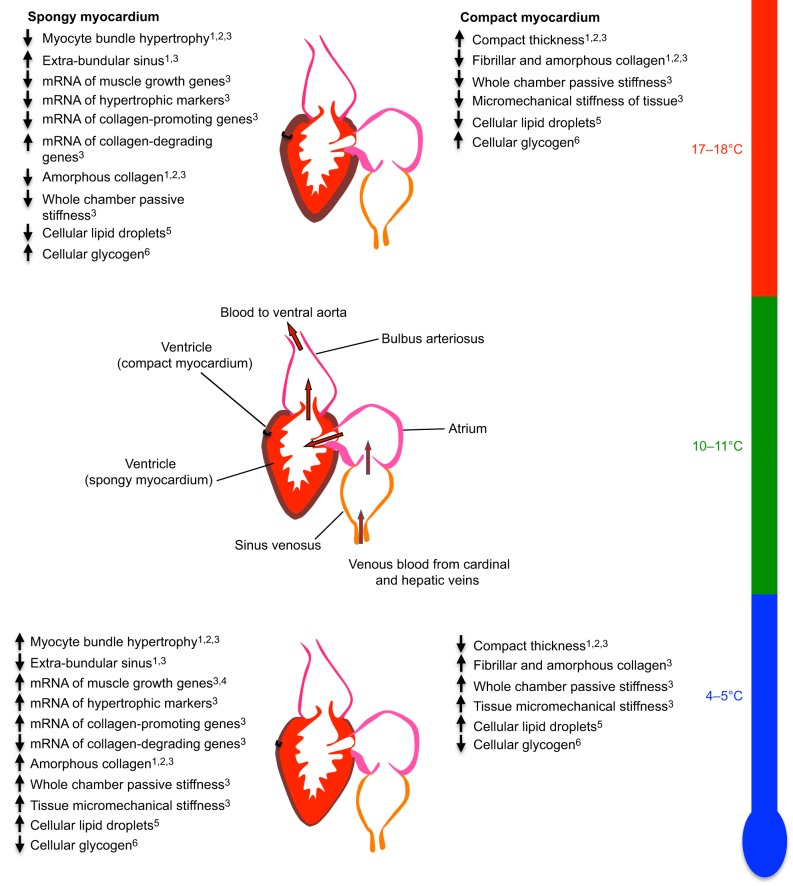
**Thermal remodelling of the rainbow trout heart.** A summary of the effects of chronic cooling (5°C) and chronic warming (18°C) on the rainbow trout heart, compared with those of fish kept at control temperature (10°C). ^1^Klaiman et al., 2011; ^2^Klaiman et al., 2014; ^3^Keen et al., 2016; ^4^Vornanen et al., 2005; ^5^Driedzic et al., 1996; ^6^Driedzic and Gesser, 1994.

Cardiac hypertrophy following cold acclimation in fish is a strategy to help compensate for the effect of low temperature on the active properties of the muscle by increasing muscle mass, thus increasing the pressure-generating ability of the myocardium (Driedzic et al., 1996; Gamperl and Farrell, 2004; Graham and Farrell, 1989; Keen et al., 2016; Klaiman et al., 2011). However, recent work by Klaiman et al. (2014) demonstrated that cold acclimation of trout can increase the pressure-generating capacity of the heart in the absence of a hypertrophic response (Fig. 5C). This change in function is likely to be due, at least in part, to alterations of the myofilaments (see ‘Myofibril remodelling’ above). In this study, there were also changes to the morphology of the heart (Klaiman et al., 2014), including a decrease in the relative proportion of compact myocardium and a reciprocal increase in spongy myocardium (Fig. 6 and Table 1). Such a change in cardiac morphology with cold acclimation has been reported in other studies of trout (Farrell et al., 1988; Keen et al., 2016; Klaiman et al., 2011), as well as for zebrafish (Johnson et al., 2014). In the fish heart, the spongy myocardium is composed of trabecular sheets that enable the formation of lacunae that fill with blood during diastole. Then, during systole, the ventricular trabeculae act as ‘contractile girders’, helping to pull the compact myocardium inwards during contraction (Pieperhoff et al., 2009). Additionally, the small lacunae that are formed by the trabecular nature of the spongy muscle lower the wall tension against which the myocytes have to work, i.e. the trabeculae reduce the cardiac work load as explained by LaPlace's law. This functional organization of the myocardium is thought to enable the extremely high ejection fraction of the trout heart (∼80%) compared with that of the mammalian heart (50–60%), which does not contain spongy myocardium (Franklin and Davie, 1992). The observed increase in spongy myocardium seen in the trout heart with cold acclimation would, therefore, increase the stroke volume of the heart while also increasing the relative proportion of contractile machinery. Such a change would make the heart able to pump more blood per contraction.

**Table 1.**
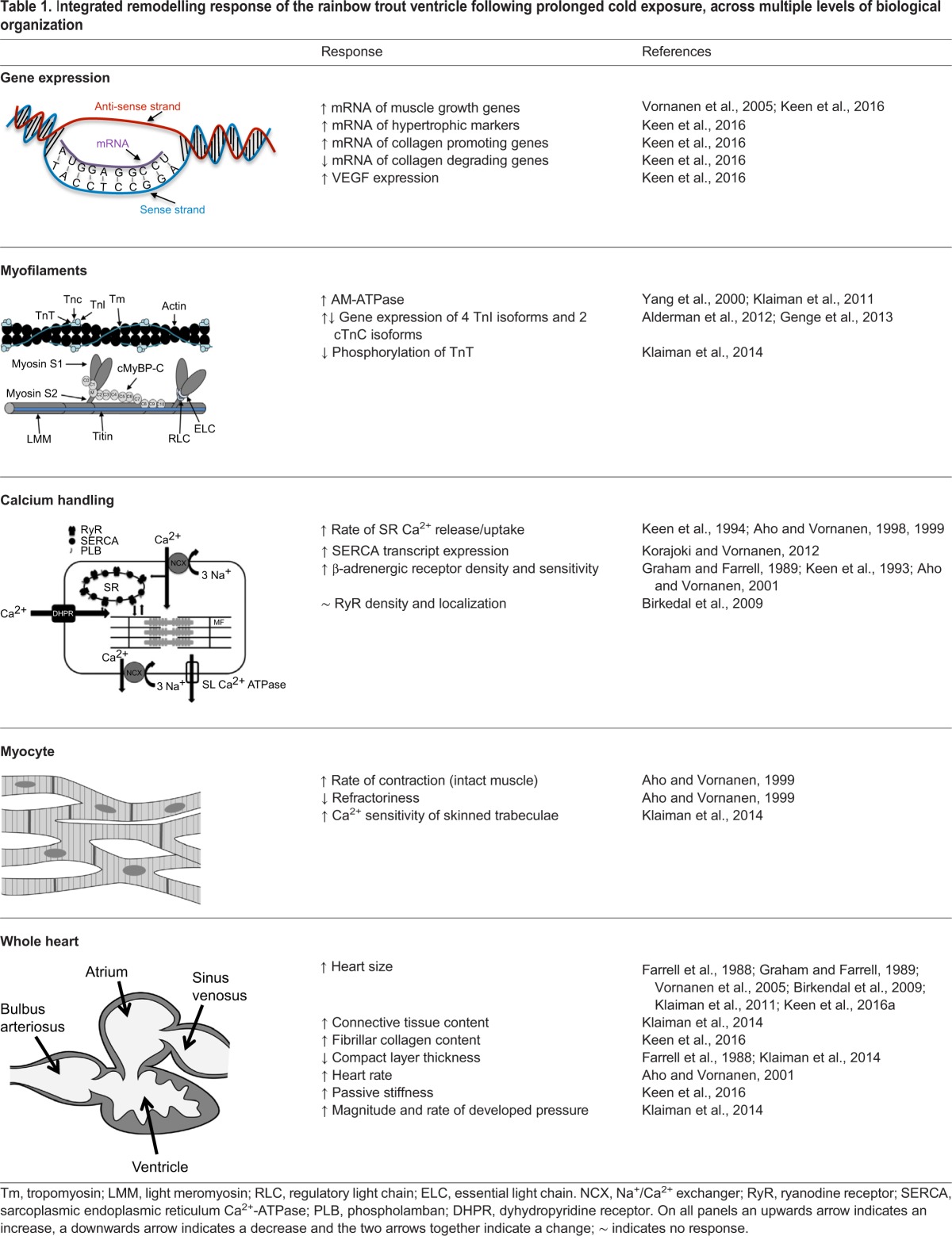
I**ntegrated remodelling response of the rainbow trout ventricle following prolonged cold exposure, across multiple levels of biological organization**

#### Length-dependent changes in force generation

Changes in the resting length of the sarcomere can affect the strength of contraction and, thus, the pressure-generating capacity of the ventricle. Interestingly, Klaiman et al. (2014) demonstrated that the difference in developed pressure at higher ventricle volumes between hearts from cold- and warm-acclimated fish was greater than at smaller ventricle volumes. One possible explanation for this result is that the cardiac muscle of fish that have been acclimated to high or low temperatures may respond differently to stretch. As discussed above, rainbow trout cardiac muscle has a larger working range of the Frank–Starling curve compared with that of rats, as well as a longer optimal sarcomere length (2.6 µm versus 2.2 µm) (Patrick, et al., 2010; Shiels et al., 2006a; Cazorla et al., 2000). In addition, previous work in mammals has shown that following a physiological stressor such as exercise training, cardiac tissue has a greater response to stretch (known as length-dependent activation) (Diffee and Nagle, 2003). Thus, it is possible that length-dependent activation is more prominent in the trout heart following acclimation to cold temperatures. This hypothesis deserves future investigation.

### Phase 3 – Ejection

The third stage of the cardiac cycle is ejection. Following pressure generation by the myocardium, the bulbo-ventricular valve opens, and blood is forced from the ventricle into the bulbus arteriosus in the fish outflow tract and from there to the rest of the body. In zebrafish, ejection time decreases with acute reductions in ambient temperature; however, there are no effects following cold acclimation (Lee et al., 2016). Heart rate determines the duration between ejections. Although an acute decrease in temperature slows heart rate (Driedzic and Gesser, 1994), cold acclimation results in partial thermal compensation (Aho and Vornanen, 1999; Little and Seebacher, 2014). The end result may increase isometric force generation and thus ejection of blood from the ventricle. Conversely, stroke volume is not altered by acute temperature change (Clark et al., 2008; Gollock et al., 2006; Lee et al., 2016; Mendonca et al., 2007; Steinhausen et al., 2008), whereas during chronic cooling it may remain constant or increase. Although Lee et al. (2016) showed that stroke volume peaks when ambient temperature matches acclimation temperature, cold acclimation significantly increases systolic function, with increases in ejection fraction and fractional shortening, which is consistent with increases in the expression of contractile proteins (as explained above) (Genge et al., 2013). In zebrafish, acute temperature change does not affect the E/A ratio, suggesting that – at all temperatures – ventricular preload, and therefore ejection fraction, is primarily determined by late diastolic filling, which is dependent on atrial contraction (Farrell and Jones, 1992; Lee et al., 2016).

## Influence of warm acclimation on the structure and function of the heart

When the temperature of ventricular trabeculae from Atlantic cod was increased from 10 to 20°C, the amount of work required to lengthen the preparations nearly doubled (Syme et al., 2013). The authors suggest that this was due to an increase in the resting tension of the muscle (Syme et al., 2013). Such a response could be caused by the increase in temperature enhancing the Ca^2+^ sensitivity of the myofilament, thereby increasing the number of active cross-bridges during diastole (Gillis et al., 2005, 2000, 2003b; Gillis and Tibbits, 2002). This effect would stiffen the muscle, impair cardiac filling and potentially limit the ability of the fish to maintain stroke volume as temperature rises (Syme et al., 2013). Therefore, just as the structure and function of the fish heart may remodel to (partially) compensate for a decrease in ambient temperature, it may also remodel to offset the effects of increased environmental temperatures. For wild fish, increases in ambient temperature may be more complex than the decreases associated with winter cold, as flow, shade and water depth can all affect water temperature. As such, behavioural thermoregulation is likely to play a key role in keeping fish cool. However, as overall ambient temperature increases with global climate change, ectothermic animals living in temperate environments are likely to experience larger temperature fluctuations, including periods of higher than average temperatures during summer months. The ability of fish species to respond to acute and prolonged changes in temperature may therefore be essential for their long-term survival.

Although laboratory-based temperature acclimation studies do not capture the complexity of temperatures that fish may encounter in open water, they offer an insight into the physiological remodelling that may occur. For example Badr et al. (2016) demonstrated that warm acclimation increases the temperature at which heart rate becomes irregular in the roach *Rutilus rutilus.* In addition, Klaiman et al. (2014) demonstrated that there is a decrease in the magnitude and rate of ventricular pressure generation in hearts from warm-acclimated trout compared with control (11°C) and cold-acclimated (4°C) fish when measured at a common experimental temperature (Figs 5 and 6). Our groups have also demonstrated that warm acclimation causes a reduction in overall ventricular mass, an increase in the thickness of the compact layer, and a decrease in connective tissue content (Klaiman et al., 2011; Keen et al., 2016). The decreased ventricular mass is attributed to a reduction in the area of the spongy myocardium; therefore, morphologically, the ventricle shows the direct opposite response to that observed following cold acclimation. The increase in compact layer thickness and decrease in spongy layer thickness are linked to a functional increase in ventricular compliance (Keen et al., 2016), suggesting that the volume of blood being pumped per beat is reduced. As an increase in physiological temperature increases heart rate in fish (Aho and Vornanen, 2001; Badr et al., 2016; Lee et al., 2016), this suggests that the heart is pumping less blood per beat at a faster rate. What is currently unknown, however, is whether and how the trout heart can remodel to temperatures above its normal seasonal range, and what the functional consequence of such remodelling is.

It is interesting to note that the cold-induced increase in collagen deposition documented in the trout heart is reversed following chronic warming (Klaiman et al., 2011; Keen et al., 2016). In contrast, in mammals collagen deposition can become relatively fixed and is often the substrate for cardiac pathologies (arrhythmias, diastolic dysfunction) in mammals (e.g. Nattel et al., 2008). Indeed, in mammalian hearts, removing or reversing the trigger for remodelling does not necessarily result in a reversion to the original state. The plasticity of the remodelling responses to warming and cooling is obviously well suited to a mesothermic fish such as the trout, but the mechanisms that permit these often opposite responses are only just beginning to be examined.

## Conclusions

The ability of some fish to remodel their heart in response to changes in environmental temperature has ecological consequences, as it enables them to remain active over a wide range of environmental temperatures. Such plasticity may also improve their ability to maintain cardiac function as average seasonal temperatures increase with global climate change. Independent of these potential advantages, the ability of fish to remodel their heart in response to changes in environmental conditions is a significant feat that results from significant phenotypic plasticity. Current and future studies should investigate how rapidly a fish heart can remodel in response to a change in environmental temperature and examine the physiological consequences of multiple remodelling events. In addition, all known studies have looked at fixed time points (6 or 8 weeks) of thermal acclimation and not at the time course of remodelling. Such information would be relevant to understanding how stochastic environmental temperatures may affect natural fish populations. Such knowledge also has significant biomedical application by increasing our understanding of what limits the ability of the vertebrate heart to remodel in response to a physiological stressor and providing novel insights useful for the development of strategies to control pathological remodelling seen in mammalian hearts.

## References

[JEB128496C1] Aho E. and Vornanen M. (1998). Ca^2+^-ATPase activity and Ca^2+^ uptake by sarcoplasmic reticulum in fish heart: effects of thermal acclimation. *J. Exp. Biol.* 201, 525-532. 9438828 10.1242/jeb.201.4.525

[JEB128496C2] Aho E. and Vornanen M. (1999). Contractile properties of atrial and ventricular myocardium of the heart of rainbow trout *Oncorhynchus mykiss*: effects of thermal acclimation. *J. Exp. Biol.* 202, 2663-2677. 10482725 10.1242/jeb.202.19.2663

[JEB128496C3] Aho E. and Vornanen M. (2001). Cold acclimation increases basal heart rate but decreases its thermal tolerance in rainbow trout (*Oncorhynchus mykiss*). *J. Comp. Physiol. B Biochem. Syst. Environ. Physiol.* 171, 173-179. 10.1007/s003600000171 11302534

[JEB128496C4] Alderman S. L., Klaiman J. M., Deck C. A. and Gillis T. E. (2012). Effect of cold acclimation on troponin I isoform expression in striated muscle of rainbow trout. *Am. J. Physiol. Regul. Integr. Comp. Physiol.* 303, R168-R176. 10.1152/ajpregu.00127.2012 22592558

[JEB128496C5] Anttila K., Couturier C. S., Øverli O., Johnsen A., Marthinsen G., Nilsson G. E. and Farrell A. P. (2014). Atlantic salmon show capability for cardiac acclimation to warm temperatures. *Nat. Commun.* 5, 4252 10.1038/ncomms5252 24957572

[JEB128496C6] Badr A., El-Sayed M. F. and Vornanen M. (2016). Effects of seasonal acclimatization on temperature dependence of cardiac excitability in the roach, *Rutilus rutilus*. *J. Exp. Biol.* 219, 1495-1504. 10.1242/jeb.138347 26994185

[JEB128496C7] Bailey J. R. and Driedzic W. R. (1990). Enhanced maximum frequency and force development of fish hearts following temperature acclimation. *J. Exp. Biol.* 149, 239-254.

[JEB128496C8] Ballesta S., Hanson L. M. and Farrell A. P. (2012). The effect of adrenaline on the temperature dependency of cardiac action potentials in pink salmon *Oncorhynchus gorbuscha*. *J. Fish Biol.* 80, 876-885. 10.1111/j.1095-8649.2011.03187.x 22471806

[JEB128496C9] Birkedal R., Christopher J., Thistlethwaite A. and Shiels H. A. (2009). Temperature acclimation has no effect on ryanodine receptor expression or subcellular localization in rainbow trout heart. *J. Comp. Physiol. B* 179, 961-969. 10.1007/s00360-009-0377-x 19544062

[JEB128496C122] Bishop S. P. (1990). The myocardial cell: normal growth, cardiac hypertrophy and response to injury. *Toxicol. Pathol.* 18, 438-453. 10.1086/318095 2151058

[JEB128496C10] Blumenschein T. M. A., Gillis T. E., Tibbits G. F. and Sykes B. D. (2004). Effect of temperature on the structure of trout troponin C. *Biochemistry* 43, 4955-4963. 10.1021/bi035504z 15109253

[JEB128496C11] Boustany A. M., Matteson R., Castleton M., Farwell C. and Block B. A. (2010). Movements of pacific bluefin tuna (*Thunnus orientalis*) in the Eastern North Pacific revealed with archival tags. *Prog. Oceanogr.* 86, 94-104. 10.1016/j.pocean.2010.04.015

[JEB128496C12] Cazorla O., Freiburg A., Helmes M., Centner T., McNabb M., Wu Y., Trombitas K., Labeit S. and Granzier H. (2000). Differential expression of cardiac titin isoforms and modulation of cellular stiffness. *Circ. Res.* 86, 59-67. 10.1161/01.RES.86.1.59 10625306

[JEB128496C13] Chandra M., Dong W.-J., Pan B.-S., Cheung H. C. and Solaro R. J. (1997). Effects of protein kinase A phosphorylation on signaling between cardiac troponin I and the N-terminal domain of cardiac troponin C. *Biochemistry* 36, 13305-13311. 10.1021/bi9710129 9341222

[JEB128496C14] Churcott C. S., Moyes C. D., Bressler B. H., Baldwin K. M. and Tibbits G. F. (1994). Temperature and pH effects on Ca^2+^ sensitivity of cardiac myofibrils: a comparison of trout with mammals. *Am. J. Physiol.* 267, R62-R70. 8048646 10.1152/ajpregu.1994.267.1.R62

[JEB128496C15] Clark R. J. and Rodnick K. J. (1999). Pressure and volume overloads are associated with ventricular hypertrophy in male rainbow trout. *Am. J. Physiol.* 277, R938-R946. 10516230 10.1152/ajpregu.1999.277.4.R938

[JEB128496C16] Clark T. D., Taylor B. D., Seymour R. S., Ellis D., Buchanan J., Fitzgibbon Q. P. and Frappell P. B. (2008). Moving with the beat: heart rate and visceral temperature of free-swimming and feeding bluefin tuna. *Proc. R. Soc. B Biol. Sci.* 275, 2841-2850. 10.1098/rspb.2008.0743 PMC2605832 18755679

[JEB128496C17] Cleutjens J. P. M. and Creemers E. E. J. M. (2002). Integration of concepts: cardiac extracellular matrix remodeling after myocardial infarction. *J. Card. Fail.* 8, S344-S348. 10.1054/jcaf.2002.129261 12555143

[JEB128496C18] Collier P., Watson C. J., van Es M. H., Phelan D., McGorrian C., Tolan M., Ledwidge M. T., McDonald K. M. and Baugh J. A. (2012). Getting to the heart of cardiac remodeling; how collagen subtypes may contribute to phenotype. *J. Mol. Cell. Cardiol.* 52, 148-153. 10.1016/j.yjmcc.2011.10.002 22008391

[JEB128496C19] Cortemeglia C. and Beitinger T. L. (2005). Temperature tolerances of wild-type and red transgenic zebra Danios. *Trans. Am. Fish. Soc.* 134, 1431-1437. 10.1577/T04-197.1

[JEB128496C117] Crockford T. and Johnston I. A. (1990). Temperature acclimation and the expression of contractile protein isoforms in the skeletal muscles of the common carp (*Cyprinus carpio L.*). *J. Comp. Physiol.* 160, 23-30. 10.1007/BF00258759

[JEB128496C20] Diffee G. M. and Nagle D. F. (2003). Exercise training alters length dependence of contractile properties in rat myocardium. *J. Appl. Physiol.* 94, 1137-1144. 10.1152/japplphysiol.00565.2002 12391046

[JEB128496C21] Dong W.-J., Jayasundar J. J., An J., Xing J. and Cheung H. C. (2007). Effects of PKA phosphorylation of cardiac troponin I and strong crossbridge on conformational transitions of the N-domain of cardiac troponin C in regulated thin filaments. *Biochemistry* 46, 9752-9761. 10.1021/bi700574n 17676764 PMC2547119

[JEB128496C22] Dorn G. W. II (2007). The fuzzy logic of physiological cardiac hypertrophy. *Hypertension* 49, 962-970. 10.1161/HYPERTENSIONAHA.106.079426 17389260

[JEB128496C23] Dorn G. W. II, Robbins J. and Sugden P. H. (2003). Phenotyping hypertrophy: eschew obfuscation. *Circ. Res.* 92, 1171-1175. 10.1161/01.RES.0000077012.11088.BC 12805233

[JEB128496C24] Driedzic W. R. and Gesser H. (1994). Energy metabolism and contractility in ectothermic vertebrate hearts: hypoxia, acidosis, and low temperature. *Physiol. Rev.* 74, 221-258. 8295934 10.1152/physrev.1994.74.1.221

[JEB128496C25] Driedzic W. R., Bailey J. R. and Sephton D. H. (1996). Cardiac Adaptations to low temperature in non-polar teleost fish. *J. Exp. Zool.* 275, 186-195. 10.1002/(SICI)1097-010X(19960601/15)275:2/3<186::AID-JEZ10>3.0.CO;2-I

[JEB128496C26] Eghbali M. and Weber K. T. (1990). Collagen and the myocardium: fibrillar structure, biosynthesis and degradation in relation to hypertrophy and its regression. *Mol. Cell. Biochem.* 96, 1-14. 10.1007/BF00228448 2146489

[JEB128496C27] Ekstrom A., Hellgren K., Grans A., Pichaud N. and Sandblom E. (2016). Dynamic changes in scope for heart rate and cardiac autonomic control during warm acclimation in rainbow trout. *J. Exp. Biol.* 219, 1106-1109. 10.1242/jeb.134312 26896548

[JEB128496C28] Elliott J. M. and Elliott J. A. (2010). Temperature requirements of Atlantic salmon *Salmo salar*, brown trout *Salmo trutta* and Arctic charr *Salvelinus alpinus*: predicting the effects of climate change. *J. Fish Biol.* 77, 1793-1817. 10.1111/j.1095-8649.2010.02762.x 21078091

[JEB128496C29] Farrell A. P. (1991). From Hagfish to Tuna: a perspective on cardiac function in fish. *Physiol. Zool.* 64, 1137-1164. 10.1086/physzool.64.5.30156237

[JEB128496C30] Farrell A. P. (2002). Cardiorespiratory performance in salmonids during exercise at high temperature: insights into cardiovascular design limitations in fishes. *Comp. Biochem. Physiol. A Mol. Integr. Physiol.* 132, 797-810. 10.1016/S1095-6433(02)00049-1 12095864

[JEB128496C31] Farrell A. P. and Jones D. R. (1992). The heart. In *Fish Physiology, The Cardiovascular System*. Vol. 12A, pp. 1-88. London: Acedemic Press Inc.

[JEB128496C32] Farrell A. P., Hammons A. M., Graham M. S. and Tibbits G. F. (1988). Cardiac growth in rainbow trout, *Salmo-Gairdneri*. *Can. J. Zool.* 66, 2368-2373. 10.1139/z88-351

[JEB128496C33] Farrell A., Gamperl A., Hicks J., Shiels H. and Jain K. (1996). Maximum cardiac performance of rainbow trout (*Oncorhynchus mykiss*) at temperatures approaching their upper lethal limit. *J. Exp. Biol.* 199, 663-672. 9318401 10.1242/jeb.199.3.663

[JEB128496C34] Fomovsky G. M. and Holmes J. W. (2010). Evolution of scar structure, mechanics, and ventricular function after myocardial infarction in the rat. *Am. J. Physiol.* 298, H221-H228. 10.1152/ajpheart.00495.2009 PMC2806135 19897714

[JEB128496C35] Franklin C. E. and Davie P. S. (1992). Dimensional analysis of the ventricle of an in situ perfused trout heart using echocardiography. *J. Exp. Biol.* 166, 47-60. 1602279 10.1242/jeb.166.1.47

[JEB128496C36] Fukuda N., Wu Y., Farman G., Irving T. C. and Granzier H. (2005). Titin-based modulation of active tension and interfilament lattice spacing in skinned rat cardiac muscle. *Pflugers Arch.* 449, 449-457. 10.1007/s00424-004-1354-6 15688246

[JEB128496C37] Fuzzen M. L. M., Van Der Kraak G. and Bernier N. J. (2010). Stirring up new ideas about the regulation of the hypothalamic-pituitary-interrenal axis in zebrafish (*Danio rerio*). *Zebrafish* 7, 349-358. 10.1089/zeb.2010.0662 21091199

[JEB128496C38] Galli G. L. J., Shiels H. A. and Brill R. W. (2009). Temperature sensitivity of cardiac function in pelagic fishes with different vertical mobilities: yellowfin tuna (*Thunnus albacares*), bigeye tuna (*Thunnus obesus*), mahimahi (*Coryphaena hippurus*), and swordfish (*Xiphias gladius*). *Physiol. Biochem. Zool.* 82, 280-290. 10.1086/597484 19284308

[JEB128496C39] Gamperl A. K. and Farrell A. P. (2004). Cardiac plasticity in fishes: environmental influences and intraspecific differences. *J. Exp. Biol.* 207, 2539-2550. 10.1242/jeb.01057 15201287

[JEB128496C40] Genge C. E., Davidson W. S. and Tibbits G. F. (2013). Adult teleost heart expresses two distinct troponin C paralogs: cardiac TnC and a novel and teleost-specific ssTnC in a chamber- and temperature-dependent manner. *Physiol. Genomics* 45, 866-875. 10.1152/physiolgenomics.00074.2013 23881286 PMC5471341

[JEB128496C41] Gillis T. E. and Klaiman J. M. (2011). The influence of PKA treatment on the Ca^2+^ activation of force generation by trout cardiac muscle. *J. Exp. Biol.* 214, 1989-1996. 10.1242/jeb.052084 21613514

[JEB128496C42] Gillis T. E. and Tibbits G. F. (2002). Beating the cold: the functional evolution of troponin C in teleost fish. *Comp. Biochem. Physiol. A Mol. Integr. Physiol.* 132, 763-772. 10.1016/S1095-6433(02)00046-6 12095861

[JEB128496C43] Gillis T. E., Marshall C. R., Xue X. H., Borgford T. J. and Tibbits G. F. (2000). Ca^2+^ binding to cardiac troponin C: effects of temperature and pH on mammalian and salmonid isoforms. *Am. J. Physiol.* 279, R1707-R1715. 10.1152/ajpregu.2000.279.5.R1707 11049853

[JEB128496C44] Gillis T. E., Blumenschein T. M. A., Sykes B. D. and Tibbits G. F. (2003a). Effect of temperature and the F27W mutation on the Ca^2+^ activated structural transition of trout cardiac troponin C. *Biochemistry* 42, 6418-6426. 10.1021/bi0340494 12767223

[JEB128496C45] Gillis T. E., Moyes C. D. and Tibbits G. F. (2003b). Sequence mutations in teleost cardiac troponin C that are permissive of high Ca^2+^ affinity of site II. *Am. J. Physiol.* 284, C1176-C1184. 10.1152/ajpcell.00339.2002 12519747

[JEB128496C46] Gillis T. E., Liang B., Chung F. and Tibbits G. F. (2005). Increasing mammalian cardiomyocyte contractility with residues identified in trout troponin C. *Physiol. Genomics* 22, 1-7. 10.1152/physiolgenomics.00007.2005 15784699

[JEB128496C47] Gollock M. J., Currie S., Petersen L. H. and Gamperl A. K. (2006). Cardiovascular and haematological responses of Atlantic cod (*Gadus morhua*) to acute temperature increase. *J. Exp. Biol.* 209, 2961-2970. 10.1242/jeb.02319 16857880

[JEB128496C116] Gordon A. M., Huxley A. F. and Julian F. J. (1966). The variation in isometric tension with sarcomere length in vertebrate muscle fibres. *J. Physiol.* 184, 170-192. 10.1113/jphysiol.1966.sp007909 5921536 PMC1357553

[JEB128496C48] Graham M. S. and Farrell A. P. (1989). The effect of temperature acclimation and adrenaline on the performance of a perfused trout heart. *Physiol. Zool.* 62, 38-61. 10.1086/physzool.62.1.30159997

[JEB128496C49] Graham M. S. and Fletcher G. L. (1985). On the low viscosity blood of two cold water, marine sculpins: a comparison with the winter flounder. *J. Comp. Physiol. B* 155, 455-459. 10.1007/BF00684675

[JEB128496C50] Granzier H., Helmes M. and Trombitás K. (1996). Nonuniform elasticity of titin in cardiac myocytes: a study using immunoelectron microscopy and cellular mechanics. *Biophys. J.* 70, 430-442. 10.1016/S0006-3495(96)79586-3 8770219 PMC1224941

[JEB128496C51] Harrison S. M. and Bers D. M. (1989). Influence of temperature on the calcium sensitivity of the myofilaments of skinned ventricular muscle from the rabbit. *J. Gen. Physiol.* 93, 411-428. 10.1085/jgp.93.3.411 2703821 PMC2216215

[JEB128496C52] Harrison S. M. and Bers D. M. (1990). Temperature dependence of myofilament Ca^2+^ sensitivity of rat, guinea pig, and frog ventricular muscle. *Am. J. Physiol.* 258, C274-C281. 2305870 10.1152/ajpcell.1990.258.2.C274

[JEB128496C53] Haverinen J. and Vornanen M. (2007). Temperature acclimation modifies sinoatrial pacemaker mechanism of the rainbow trout heart. *Am. J. Physiol.* 292, R1023-R1032. 10.1152/ajpregu.00432.2006 17008459

[JEB128496C54] Helmes M., Trombitas K. and Granzier H. (1996). Titin develops restoring force in rat cardiac myocytes. *Circ. Res.* 79, 619-626. 10.1161/01.RES.79.3.619 8781495

[JEB128496C55] Hiesinger W., Brukman M. J., McCormick R. C., Fitzpatrick J. R. III, Frederick J. R., Yang E. C., Muenzer J. R., Marotta N. A., Berry M. F., Atluri P. et al. (2012). Myocardial tissue elastic properties determined by atomic force microscopy after stromal cell-derived factor 1alpha angiogenic therapy for acute myocardial infarction in a murine model. *J. Thorac. Cardiovasc. Surg.* 143, 962-966. 10.1016/j.jtcvs.2011.12.028 22264415 PMC4155937

[JEB128496C56] Horowits R., Maruyama K. and Podolsky R. J. (1989). Elastic behavior of connectin filaments during thick filament movement in activated skeletal muscle. *J. Cell Biol.* 109, 2169-2176. 10.1083/jcb.109.5.2169 2808523 PMC2115863

[JEB128496C57] Hove-Madsen L. and Tort L. (1998). L-type Ca^2+^ current and excitation-contraction coupling in single atrial myocytes from rainbow trout. *Am. J. Physiol.* 275, R2061-R2069. 9843898 10.1152/ajpregu.1998.275.6.R2061

[JEB128496C58] Hu N., Yost H. J. and Clark E. B. (2001). Cardiac morphology and blood pressure in the adult zebrafish. *Anat. Rec.* 264, 1-12. 10.1002/ar.1111 11505366

[JEB128496C59] Jalil J. E., Doering C. W., Janicki J. S., Pick R., Clark W. A., Abrahams C. and Weber K. T. (1988). Structural vs. contractile protein remodeling and myocardial stiffness in hypertrophied rat left ventricle. *J. Mol. Cell. Cardiol.* 20, 1179-1187. 10.1016/0022-2828(88)90597-4 2470910

[JEB128496C60] Jalil J. E., Doering C. W., Janicki J. S., Pick R., Shroff S. G. and Weber K. T. (1989). Fibrillar collagen and myocardial stiffness in the intact hypertrophied rat left ventricle. *Circ. Res.* 64, 1041-1050. 10.1161/01.RES.64.6.1041 2524288

[JEB128496C61] Johnson A. C., Turko A. J., Klaiman J. M., Johnston E. F. and Gillis T. E. (2014). Cold acclimation alters the connective tissue content of the zebrafish (Danio rerio) heart. *J. Exp. Biol.* 217, 1868-1875. 10.1242/jeb.101196 24577447

[JEB128496C62] Jørgensen S. M., Castro V., Krasnov A., Torgersen J., Timmerhaus G., Hevrøy E. M., Hansen T. J., Susort S., Breck O. and Takle H. (2014). Cardiac responses to elevated seawater temperature in Atlantic salmon. *BMC Physiol.* 14, 2 10.1186/1472-6793-14-2 24581386 PMC3944800

[JEB128496C63] Katz A. M. (2006). *Physiology of the Heart*. Philadelphia: Lippincott Williams & Wilkins.

[JEB128496C64] Keen J. E., Vianzon D.-E., Farrell A. P. and Tibbits G. F. (1993). Thermal acclimationalters both adrenergic sensitivity and adrenoceptor density in cardiac tissue of rainbow trout. *J. Exp. Biol.* 181, 27-47.

[JEB128496C65] Keen J. E., Vianzon D.-M., Farrell A. P. and Tibbits G. F. (1994). Effect of temperature and temperature acclimation on the ryanodine sensitivity of the trout myocardium. *J. Comp. Physiol. B* 164, 438-443. 10.1007/BF00714580

[JEB128496C66] Keen A. N., Fenna A. J., McConnell J. C., Sherratt M. J., Gardner P. and Shiels H. A. (2016). The dynamic nature of hypertrophic and fibrotic remodeling of the fish ventricle. *Front. Physiol.* 6, 427 10.3389/fphys.2015.00427 26834645 PMC4720793

[JEB128496C67] Kent J., Koban M. and Prosser C. L. (1988). Cold-acclimation-induced protein hypertrophy in channel catfish and green sunfish. *J. Comp. Physiol. B* 158, 185-198. 10.1007/BF01075832 3170825

[JEB128496C68] Klaiman J. M., Fenna A. J., Shiels H. A., Macri J. and Gillis T. E. (2011). Cardiac remodeling in fish: strategies to maintain heart function during temperature change. *PLoS ONE* 6, e24464 10.1371/journal.pone.0024464 21915331 PMC3168507

[JEB128496C69] Klaiman J. M., Pyle W. G. and Gillis T. E. (2014). Cold acclimation increases cardiac myofilament function and ventricular pressure generation in trout. *J. Exp. Biol.* 217, 4132-4140. 10.1242/jeb.109041 25278471

[JEB128496C70] Korajoki H. and Vornanen M. (2012). Expression of SERCA and phospholamban in rainbow trout (*Oncorhynchus mykiss*) heart: comparison of atrial and ventricular tissue and effects of thermal acclimation. *J. Exp. Biol.* 215, 1162-1169. 10.1242/jeb.065102 22399661

[JEB128496C71] Krüger M. and Linke W. A. (2009). Titin-based mechanical signalling in normal and failing myocardium. *J. Mol. Cell. Cardiol.* 46, 490-498. 10.1016/j.yjmcc.2009.01.004 19639676

[JEB128496C72] Lee L., Genge C. E., Cua M., Sheng X., Rayani K., Beg M. F., Sarunic M. V. and Tibbits G. F. (2016). Functional assessment of cardiac responses of adult Zebrafish (*Danio rerio*) to acute and chronic temperature change using high-resolution echocardiography. *PLoS ONE* 11, e0145163 10.1371/journal.pone.0145163 26730947 PMC4701665

[JEB128496C73] Linke W. A. (2008). Sense and stretchability: the role of titin and titin-associated proteins in myocardial stress-sensing and mechanical dysfunction. *Cardiovasc. Res.* 77, 637-648. 17475230 10.1016/j.cardiores.2007.03.029

[JEB128496C74] Linke W. A., Ivemeyer M., Olivieri N., Kolmerer B., Rüegg C. J. and Labeit S. (1996). Towards a molecular understanding of the elasticity of titin. *J. Mol. Biol.* 261, 62-71. 10.1006/jmbi.1996.0441 8760502

[JEB128496C75] Little A. G. and Seebacher F. (2014). Thyroid hormone regulates cardiac performance during cold acclimation in zebrafish (*Danio rerio*). *J. Exp. Biol.* 217, 718-725. 10.1242/jeb.096602 24265422

[JEB128496C76] Liu B., Wohlfart B. and Johansson B. W. (1990). Effects of low temperature on contraction in papillary muscles from rabbit, rat, and hedgehog. *Cryobiology* 27, 539-546. 10.1016/0011-2240(90)90041-2 2249456

[JEB128496C77] Liu B., Wang L. C. and Belke D. D. (1993). Effects of temperature and pH on cardiac myofilament Ca^2+^ sensitivity in rat and ground squirrel. *Am. J. Physiol.* 264, R104-R108. 8430869 10.1152/ajpregu.1993.264.1.R104

[JEB128496C78] López-Olmeda J. F. and Sánchez-Vázquez F. J. (2011). Thermal biology of zebrafish (*Danio rerio*). *J. Ther. Biol.* 36, 91-104. 10.1016/j.jtherbio.2010.12.005

[JEB128496C79] Lurman G. J., Petersen L. H. and Gamperl A. K. (2012). In situ cardiac performance of Atlantic cod (*Gadus morhua*) at cold temperatures: long-term acclimation, acute thermal challenge and the role of adrenaline. *J. Exp. Biol.* 215, 4006-4014. 10.1242/jeb.069849 22899537

[JEB128496C80] Marijianowski M. M. H., Teeling P., Mann J. and Becker A. E. (1995). Dilated cardiomyopathy is associated with an increase in the type I/type III collagen ratio: a quantitative assessment. *J. Am. Col. Cardiol.* 25, 1263-1272. 10.1016/0735-1097(94)00557-7 7722119

[JEB128496C81] Mendonca P. C., Genge A. G., Deitch E. J. and Gamperl A. K. (2007). Mechanisms responsible for the enhanced pumping capacity of the in situ winter flounder heart (*Pseudopleuronectes americanus*). *Am. J. Physiol.* 293, R2112-R2119. 10.1152/ajpregu.00202.2007 17761512

[JEB128496C82] Mutungi G. and Ranatunga K. W. (1998). Temperature-dependent changes in the viscoelasticity of intact resting mammalian (rat) fast- and slow-twitch muscle fibres. *J. Physiol.* 508, 253-265. 10.1111/j.1469-7793.1998.253br.x 9490847 PMC2230871

[JEB128496C120] Nadal-Ginard B., Kajstura J., Leri A. and Anversa P. (2003). Myocyte death, growth, and regeneration in cardiac hypertrophy and failure. *Circ. Res.* 92, 139-150. 10.1161/01.RES.0000053618.86362.DF 12574141

[JEB128496C83] Nattel S., Burstein B. and Dobrev D. (2008). Atrial remodeling and atrial fibrillation: mechanisms and implications. *Circulation* 1, 62-73. 10.1161/CIRCEP.107.754564 19808395

[JEB128496C84] Neagoe C., Kulke M., del Monte F., Gwathmey J. K., de Tombe P. P., Hajjar R. J. and Linke W. A. (2002). Titin isoform switch in ischemic human heart disease. *Circulation* 106, 1333-1341. 10.1161/01.CIR.0000029803.93022.93 12221049

[JEB128496C85] Nelson J. (2006). *Fishes of the World*. Hoboken, NJ: Wiley.

[JEB128496C86] Patrick S. M., Hoskins A. C., Kentish J. C., White E., Shiels H. A. and Cazorla O. (2010). Enhanced length-dependent Ca^2+^ activation in fish cardiomyocytes permits a large operating range of sarcomere lengths. *J. Mol. Cell. Cardiol.* 48, 917-924. 10.1016/j.yjmcc.2010.02.008 20170661

[JEB128496C87] Pauschinger M., Knopf D., Petschauer S., Doerner A., Poller W., Schwimmbeck P. L., Kuhl U. and Schultheiss H.-P. (1999). Dilated cardiomyopathy is associated with significant changes in collagen type I/III ratio. *Circulation* 99, 2750-2756. 10.1161/01.CIR.99.21.2750 10351968

[JEB128496C88] Peng J., Raddatz K., Molkentin J. D., Wu Y., Labeit S., Granzier H. and Gotthardt M. (2007). Cardiac hypertrophy and reduced contractility in hearts deficient in the titin kinase region. *Circulation* 115, 743-751. 10.1161/CIRCULATIONAHA.106.645499 17261657

[JEB128496C89] Pieperhoff S., Bennett W. and Farrell A. P. (2009). The intercellular organization of the two muscular systems in the adult salmonid heart, the compact and the spongy myocardium. *J. Anat.* 215, 536-547. 10.1111/j.1469-7580.2009.01129.x 19627390 PMC2780571

[JEB128496C90] Rodnick K. J., Gamperl A. K., Lizars K. R., Bennett M. T., Rausch R. N. and Keeley E. R. (2004). Thermal tolerance and metabolic physiology among redband trout populations in south-eastern Oregon. *J. Fish Biol.* 64, 310-335. 10.1111/j.0022-1112.2004.00292.x

[JEB128496C91] Saito M., Takenouchi Y., Kunisaki N. and Kimura S. (2001). Complete primary structure of rainbow trout type I collagen consisting of alpha1(I)alpha2(I)alpha3(I) heterotrimers. *Eur. J. Biochem.* 268, 2817-2827. 10.1046/j.1432-1327.2001.02160.x 11358497

[JEB128496C92] Sanchez-Quintana D., Garcia-Martinez V., Climent V. and Hurle J. M. (1995). Morphological analysis of the fish heart ventricle: myocardial and connective tissue architecture in teleost species. *Ann. Anat.* 177, 267-274. 10.1016/S0940-9602(11)80198-6 7541184

[JEB128496C93] Shaffer J. F. and Gillis T. E. (2010). Evolution of the regulatory control of vertebrate striated muscle: the roles of troponin I and myosin binding protein-C. *Physiol. Genomics* 42, 406-419. 10.1152/physiolgenomics.00055.2010 20484158

[JEB128496C118] Shiels H. A. and Galli G. L. (2014). The sarcoplasmic reticulum and the evolution of the vertebrate heart. *Physiology* 29, 456-469. 10.1152/physiol.00015.2014 25362639

[JEB128496C94] Shiels H. A. and White E. (2005). Temporal and spatial properties of cellular Ca^2+^ flux in trout ventricular myocytes. *Am. J. Physiol. Regul. Integr. Comp. Physiol.* 288, R1756-R1766. 10.1152/ajpregu.00510.2004 15650128

[JEB128496C95] Shiels H. A. and White E. (2008). The Frank-Starling mechanism in vertebrate cardiac myocytes. *J. Exp. Biol.* 211, 2005-2013. 10.1242/jeb.003145 18552289

[JEB128496C96] Shiels H. A., Freund E. V., Farrell A. P. and Block B. A. (1999). The sarcoplasmic reticulum plays a major role in isometric contraction in atrial muscle of yellowfin tuna. *J. Exp. Biol.* 202, 881-890. 10069977 10.1242/jeb.202.7.881

[JEB128496C97] Shiels H. A., Vornanen M. and Farrell A. P. (2000). Temperature-dependence of L-type Ca^2+^ channel current in atrial myocytes from rainbow trout. *J. Exp. Biol.* 203, 2771-2780. 10952877 10.1242/jeb.203.18.2771

[JEB128496C98] Shiels H. A., Vornanen M. and Farrell A. P. (2002). Effects of temperature on intracellular Ca^2+^ in trout atrial myocytes. *J. Exp. Biol.* 205, 3641-3650. 12409490 10.1242/jeb.205.23.3641

[JEB128496C99] Shiels H. A., Calaghan S. C. and White E. (2006a). The cellular basis for enhanced volume-modulated cardiac output in fish hearts. *J. Gen. Physiol.* 128, 37-44. 10.1085/jgp.200609543 16769795 PMC2151555

[JEB128496C100] Shiels H. A., Paajanen V. and Vornanen M. (2006b). Sarcolemmal ion currents and sarcoplasmic reticulum Ca^2+^ content in ventricular myocytes from the cold stenothermic fish, the burbot (*Lota lota*). *J. Exp. Biol.* 209, 3091-3100. 10.1242/jeb.02321 16888058

[JEB128496C101] Shiels H. A., Di Maio A., Thompson S. and Block B. A. (2011). Warm fish with cold hearts: thermal plasticity of excitation-contraction coupling in bluefin tuna. *Proc. R. Soc. B Biol. Sci.* 278, 18-27. 10.1098/rspb.2010.1274 PMC2992732 20667881

[JEB128496C102] Shiels H. A., Galli G. L. J. and Block B. A. (2015). Cardiac function in an endothermic fish: cellular mechanisms for overcoming acute thermal challenges during diving. *Proc. R. Soc. B Biol. Sci.* 282, 20141989 10.1098/rspb.2014.1989 PMC4298204 25540278

[JEB128496C103] Sidhu R., Anttila K. and Farrell A. P. (2014). Upper thermal tolerance of closely related *Danio* species. *J. Fish Biol.* 84, 982-995. 10.1111/jfb.12339 24689673

[JEB128496C104] Steinhausen M. F., Sandblom E., Eliason E. J., Verhille C. and Farrell A. P. (2008). The effect of acute temperature increases on the cardiorespiratory performance of resting and swimming sockeye salmon (*Oncorhynchus nerka*). *J. Exp. Biol.* 211, 3915-3926. 10.1242/jeb.019281 19043063

[JEB128496C105] Stephenson D. G. and Williams D. A. (1985). Temperature-dependent calcium sensitivity changes in skinned muscle fibres of rat and toad. *J. Physiol.* 360, 1-12. 10.1113/jphysiol.1985.sp015600 3921690 PMC1193444

[JEB128496C106] Sun X., Hoage T., Bai P., Ding Y., Chen Z., Zhang R., Huang W., Jahangir A., Paw B., Li Y.-G. et al. (2009). Cardiac hypertrophy involves both myocyte hypertrophy and hyperplasia in anemic zebrafish. *PLoS ONE* 4, e6596 10.1371/journal.pone.0006596 19672293 PMC2719798

[JEB128496C107] Syme D. A., Gamperl A. K., Nash G. W. and Rodnick K. J. (2013). Increased ventricular stiffness and decreased cardiac function in Atlantic cod (*Gadus morhua*) at high temperatures. *Am. J. Physiol.* 305, R864-R876. 10.1152/ajpregu.00055.2013 23883672

[JEB128496C108] Trombitas K., Wu Y., Labeit D., Labeit S. and Granzier H. (2001). Cardiac titin isoforms are coexpressed in the half-sarcomere and extend independently. *Am. J. Physiol.* 281, H1793-H1799. 10.1152/ajpheart.2001.281.4.H1793 11557573

[JEB128496C119] Vornanen M. (1994). Seasonal and temperature-induced changes in myosin heavy chain composition of crucian carp hearts. *Am. J. Physiol.* 267, R1567-R1573. 7810767 10.1152/ajpregu.1994.267.6.R1567

[JEB128496C109] Vornanen M. (1998). L-type Ca^2+^ current in fish cardiac myocytes: effects of thermal acclimation and beta-adrenergic stimulation. *J. Exp. Biol.* 201, 533-547. 9438829 10.1242/jeb.201.4.533

[JEB128496C110] Vornanen M. (2016). The temperature dependence of electrical excitability in fish hearts. *J. Exp. Biol.* 219, 1941-1952. 10.1242/jeb.128439 27385752

[JEB128496C121] Vornanen M., Shiels H. A. and Farrell A. P. (2002). Plasticity of excitation-contraction coupling in fish cardiac myocytes. *Comp. Biochem. Physiol. A* 132, 827-846. 10.1016/S1095-6433(02)00051-X 12095866

[JEB128496C111] Vornanen M., Hassinen M., Koskinen H. and Krasnov A. (2005). Steady-state effects of temperature acclimation on the transcriptome of the rainbow trout heart. *Am. J. Physiol.* 289, R1177-R1184. 10.1152/ajpregu.00157.2005 15932967

[JEB128496C112] Watanabe K., Nair P., Labeit D., Kellermayer M. S. Z., Greaser M., Labeit S. and Granzier H. (2002). Molecular mechanics of cardiac titin's PEVK and N2B spring elements. *J. Biol. Chem.* 277, 11549-11558. 10.1074/jbc.M200356200 11799131

[JEB128496C113] Weber K. T. and Janicki J. S. (1989). Angiotensin and the remodelling of the myocardium. *Br. J. Clin. Pharmacol.* 28, 149S-150S. 10.1111/j.1365-2125.1989.tb03589.x PMC1379855 2690905

[JEB128496C114] Wu Y., Cazorla O., Labeit D., Labeit S. and Granzier H. (2000). Changes in titin and collagen underlie diastolic stiffness diversity of cardiac muscle. *J. Mol. Cell. Cardiol.* 32, 2151-2162. 10.1006/jmcc.2000.1281 11112991

[JEB128496C115] Yang H., Velema J., Hedrick M. S., Tibbits G. F. and Moyes C. D. (2000). Evolutionary and Physiological Variation in Cardiac Troponin C in Relation to Thermal Strategies of Fish. *Physiol. Biochem. Zool.* 73, 841-849. 10.1086/318095 11121357

